# Supplementary material for: Physicochemical Properties of Lipoproteins Assessed by Nuclear Magnetic Resonance as a Predictor of Premature Cardiovascular Disease. PRESARV-SEA Study
Source: J Clin Med. 2021 Mar 29;10(7):1379. doi: 10.3390/jcm10071379 (PMC8037702; doi:10.3390/jcm10071379)
Supplement: Supplementary file 1 [file jcm-10-01379-s001.pdf]

Journal of
Clinical Medicine

MDPI

Supplementary materials

Table S1. Univariate analysis.

Variables (Units)	*p* value
**Very Low Density Lipoprotein**	
Cholesterol (mg/dL)	0.309
Triglycerides (mg/dL)	0.139
Total particles (nmol/L)	0.365
Large particles (nmol/L)	0.001
Medium particles (nmol/L)	0.114
Small particles (nmol/L)	0.473
Diameter (nm)	3.38×10^{-8}
**Intermediate Density Lipoprotein**	
Cholesterol (mg/dL)	0.172
Triglycerides (mg/dL)	0.534
**Low Density Lipoprotein**	
Cholesterol (mg/dL)	2.90×10^{-13}
Triglycerides (mg/dL)	6.23×10^{-12}
Total particles (nmol/L)	7.31×10^{-13}
Large particles (nmol/L)	2.60×10^{-12}
Medium particles (nmol/L)	2.41×10^{-13}
Small particles (nmol/L)	1.30×10^{-8}
Cholesterol small particles (mmol/L)	0.001
Diameter (nm)	1.02×10^{-9}
**High Density Lipoprotein**	
Cholesterol (mg/dL)	0.024
Triglycerides (mg/dL)	1.35×10^{-25}
Total particles (μmol/L)	0.582
Large particles (μmol/L)	3.25×10^{-11}
Medium particles (μmol/L)	2.95×10^{-7}
Small particles (μmol/L)	0.001
Diameter (μmol/L)	1.30×10^{-10}

Data are expressed as medians (interquartile interval.
